# Violence risk prediction in psychiatry

**DOI:** 10.1192/j.eurpsy.2026.10409

**Published:** 2026-07-31

**Authors:** S. Fazel

**Affiliations:** Department of Psychiatry, University of Oxford, Oxford, United Kingdom

## Abstract

**Abstract:**

Across medicine, accurate prognosis can inform clinical decision-making about management and resource allocation. Several such models have been developed and validated in psychiatry, including those estimating risk of psychosis onset and relapse, and other key adverse outcomes, such as suicide and violence. Reliable risk estimation for these outcomes could inform follow-up intensity, continuity of care, collaborative safety planning, and risk management.

This presentation will provide an overview of prognostic models for violence in people with mental illness. I will outline the predictive performance, acceptability, and impact of new models, which have been translated into clinical risk assessment tools. I will show promising findings for these new models, which could be included in clinical guidelines. Further research is required to study whether and how they supplement and augment decision-making in different clinical settings.

**Disclosure of Interest:**

None Declared

